# A survey on the use of mice, pigs, dogs and monkeys as animal models in biomedical research in Spain

**DOI:** 10.1186/s42826-022-00124-5

**Published:** 2022-06-02

**Authors:** Olatz Goñi-Balentziaga, Iván Ortega-Saez, Sergi Vila, Garikoitz Azkona

**Affiliations:** 1 Department of Clinical and Health Psychology and Research Methodology, Euskal Herriko Unibertsitatea (UPV/EHU), Donostia, Spain; 2 Scientific and Technological Centers (CCIT), University of Barcelona (UB), Barcelona, Spain; 3 Department of Basic Psychological Processes and Their Development, Euskal Herriko Unibertsitatea (UPV/EHU), Donostia, Spain

**Keywords:** Laboratory animal, Biomedical research, Moral status, Phylogenetic scale

## Abstract

**Background:**

The use of animals in biomedical science remains controversial. An individual’s level of concern is generally influenced by their culture, previous or current experience with animals, and the specific animal species in question. In this study we aimed to explore what people in Spain who had never or who no longer worked with laboratory animals thought of the use of mice, pigs, dogs and monkeys for biomedical research purposes. We also aimed to determine whether or not people currently involved in biomedical research with the aforementioned species felt their work was justified.

**Results:**

The study comprised a total of 807 participants (never worked = 285, used to work = 56, currently working = 466), almost two thirds of whom were women. Our results revealed that the phylogenetic scale is an important factor in people’s opinions of the use of certain species in research. The percentage of people who were against the use of dogs or monkeys was higher than that of those who were against the use of mice or pigs. The main reasons given for having stopped working with laboratory animals were change of professional career and change in research project. Participants who were currently working with animals believed that their work was justified, but said they did not talk about it with people outside their immediate social circle.

**Conclusions:**

Our findings suggest that there is a difference in moral status between monkeys and mice, as well as between companion animals (dogs) and farm animals (pigs). Our results support the idea that working with laboratory animals is a sensitive issue in Spain.

**Supplementary Information:**

The online version contains supplementary material available at 10.1186/s42826-022-00124-5.

## Background

Biomedical research aims to generate benefits for human health. However, whereas almost everyone would approve the goal of relieving human suffering, the use of human beings in research experiments is generally deemed morally unacceptable. Moreover, international ethical standards for human research state that medical research involving humans must be based on previous results obtained in animal experimentation [1].

In 2013, Spain transposed Directive 2010/63/EU into its legislative framework (RD 53/2013). Consequently, Spanish legislation for the protection of animals used for scientific purposes is now based on replacement, reduction and refinement (the 3Rs). These principles promote the replacement of animals with non-sentient alternatives, the reduction of animal use, and the refinement of experiments and husbandry conditions so as to cause minimum pain and distress [2]. This constitutes a theoretical step forward in strengthening the general position on animal welfare. Currently, scientists are working to produce valid data on how to measure welfare in all laboratory animal species. To this end, they are asking for more public resources and interdisciplinary teams to solve the quandary of how to strike a balance between animal welfare legislation and scientific freedom [3]. Similarly, the Eurogroup for Animal Welfare is currently focusing its efforts on the implementation of reduction and refinement methods in research [4].

Public opinion on the use of laboratory animals is influenced by a myriad of biological and sociocultural factors [5]. For example, attitudes towards the use of animals by humans may be affected by a person's previous or current experience with animals [6, 7], and the level of concern also differs across European countries, as well as in accordance with the specific animal species in question. According to the Special Eurobarometer carried out in 2010 [8], two out of three (66%) European citizens felt that experimentation using mice was acceptable if this led to an improvement in human health and wellbeing. However, only 44% agreed (as opposed to 37% who disagreed) with the use of dogs or monkeys for the same purpose. Moreover, 75% of Spanish citizens considered the use of mice in science to be acceptable, and 65% approved of the use of dogs and monkeys. A more recent study found that people in Spain were more concerned about laboratory animal welfare than their counterparts in Romania, Lithuania, Poland and Sweden [9], and we have recently reported that people working with laboratory rodents in our country are highly aware of and sensitive to their wellbeing [10].

In 2015, a European Citizens’ Initiative (ECI) entitled “Stop Vivisection” gathered 1.17 million signatures calling for the phasing out of all animal experiments for scientific purposes [11]. Although current European legislation establishes the general objective of fully replacing all procedures that use live animals for scientific and educational purposes as soon as it is scientifically possible to do so, we are still a long way from achieving this goal. According to a report published by the Netherlands National Committee for the protection of animals used for scientific purposes (NCad), for regulatory safety testing, testing on animals could be phased out by 2025 [12]. However, in a recent referendum, Switzerland rejected a ban on animal testing [13].

The use of animals in biomedical research remains controversial. On the one hand, animal models are deemed necessary to better understanding the pathophysiology of some human diseases, since certain phenomena can only be studied in vivo [14]. Moreover, the European Medicines Agency (EMA) continues to view preclinical studies in animals as central to the medical product approval process, and is therefore unlikely to allow any scientists to embark on a clinical trial without some in vivo evidence of safety and efficacy. On the other side of the argument, however, the low degree of similarity between animals and humans calls the legitimacy of experimenting with (some) animals into question [14]. In this regard, three broad stances have been identified among society: (1) human beings have a moral importance that other animals lack; (2) a sliding moral scale exits in which humans are at the top; and (3) human beings and animals are moral equals [15].

In this study we aimed to explore how (a) people who had never worked and (b) people who no longer worked with laboratory animals viewed the use of mice, pigs, dogs and monkeys in biomedical research. We also aimed to determine whether or not (c) people involved in biomedical research with the aforementioned species believed their work was justified.

## Results

### Participant information

A total of 811 individuals started the survey, but 4 of them did not consent to being included in the study. The final sample therefore comprised a total of 807 participants from different parts of Spain (Additional file 1: Table S1), with almost two thirds being women. The personal information pertaining to each group of participants; (a) never worked, (b) used to work, and (c) currently working with laboratory animals is summarized in Table 1.

**Table 1:** Participants’ demographic information

	Never worked	Used to work	Currently working	Total
	n (%)			
*Gender*				
Cis/trans women	169 (59.3%)	31 (55.4%)	316 (67.8%)	516 (63.9%)
Cis/trans men	104 (36.5%)	24 (42.9%)	138 (29.6%)	266 (33.0%)
Non binary	2 (0.7%)	–	–	2 (0.2%)
Preferred not to say	10 (3.5%)	1 (1.8%)	12 (2.6%)	23 (2.9%)
*Age range*				
20–29	77 (27.0%)	8 (14.3%)	132 (28.3%)	217 (26.9%)
30–39	72 (25.3%)	21 (37.5%)	137 (29.4%)	230 (28.5%)
40–49	83 (29.1%)	18 (32.1%)	126 (27%)	227 (28.1%)
50–59	39 (13.7%)	8 (14.3%)	60 (12.9%)	107 (13.3%)
≥ 60	14 (4.9%)	1 (1.8%)	11 (2.4%)	26 (3.2%)
*Education*				
Primary school	5 (1.8%)	–	3 (0.6%)	8 (1%)
Secondary school	14 (4.9%)	1 (1.8%)	18 (3.9%)	33 (4.1%)
Vocational training	41 (14.4%)	8 (14.3%)	71 (15.2%)	120 (14.9%)
Undergraduate degree	190 (66.7%)	20 (35.7%)	199 (42.7%)	409 (50.7%)
PhD	35 (12.3%)	27 (48.2%)	175 (37.5%)	237 (29.4%)
Total (n)	285	56	466	807

Participants who used to work with animals were mostly researchers who had worked with them during their PhD, and less than a fifth worked as animal facility personnel. On average, they had worked for 5.5 ± 6.5 years with laboratory animals, spending 17.2 ± 14.5 h per week with them. The vast majority had worked with animals at research institutes or universities (Table 2). Most (51/91.1%) had worked with rodents (mice, rats, guinea pigs), 8 (14.3%) had worked with aquatic species, 5 (8.9%) with small carnivores (dogs, cats, ferrets) or farm animals (pigs, cows, sheep) and one participant (1.8%) had worked with non-human primates.

**Table 2:** Participants’ professional information

	Used to work	Currently working	Total
	n (%)		
*Job category*			
Welfare officer and/or veterinarian	1 (1.8%)	62 (13.3%)	63 (12.1%)
Animal caretaker or technician	9 (16.1%)	95 (20.4%)	104 (19.9%)
Researcher	15 (26.8%)	153 (32.8%)	168 (32.2%)
Research technician	8 (14.3%)	67 (14.4%)	75 (14.4%)
PhD student	23 (41.1%)	89 (19.1%)	112 (21.5%)
*Type of institution*			
University	22 (39.3%)	133 (28.5%)	155 (29.7%)
Research institute	31 (55.4%)	232 (49.8%)	263 (50.4%)
Hospital	2 (3.6%)	42 (9%)	44 (8.4%)
Contracted research organization	–	40 (8.6%)	40 (7.7%)
Pharmaceutical company	–	10 (2.1%)	10 (1.9%)
Other type of private company	1 (1.8%)	9 (1.9%)	10 (1.9%)
Total (n)	56	466	

Almost half claimed to have stopped working with animals because of a change in their professional career, although empathy towards animals, salary and change of research project were also reasons given (Table 3).

**Table 3:** Reasons for stopping working with animals, by job category

	Job category					
	Welfare officer and/or veterinarian	Animal caretaker or technician	Researcher	Research technician	PhD student	Total
Reason	n (%)					n (%)
Change of professional career	1 (100%)	7 (43.8%)	8 (47.1%)	4 (50%)	16 (50%)	36 (48.6%)
Change of research project	–	–	6 (35.3%)	3 (37.5%)	4 (12.5%)	13 (17.6%)
Empathy towards animals	–	2 (12.5%)	1 (5.9%)	–	6 (18.8%)	9 (12.2%)
Salary	–	5 (31.3%)	1 (5.9%)	–	1 (3.1%)	7 (9.5%)
Ethical issues	–	2 (12.5%)	–	–	3 (9.4%)	5 (6.8%)
Working hours	–	–	–	1 (12.5%)	1 (3.1%)	2 (2.7%)
Allergies	–	–	–	–	1 (3.1%)	1 (1.4%)
Increasing obstacles imposed by the administration	–	–	1 (5.9%)	–	–	1 (1.4%)

More than half of those who were currently working with animals were researchers (309/66.3%), and the rest worked at animal facilities (157/33.7%). On average, they claimed to have worked with animals for 11.5 ± 8.8 years, spending 16.7 ± 14.6 h per week with them. Most worked in research institutes or universities (Table 2) and the vast majority worked with mice, followed by pigs, dogs and monkeys. Upon analyzing the distribution of the different species by institution, we found that most mice were housed in the animal facilities of research centers or universities, while dogs and monkeys were mainly housed in facilities belonging to contracted research organizations (Table 4).

**Table 4:** Species worked with, by type of institution

	Mice	Pigs	Dogs	Monkeys
*Institution*				
University	121 (33.4%)	10 (10.6%)	–	1 (4.3%)
Research institute	206 (56.9%)	27 (28.7%)	2 (8.7%)	–
Hospital	24 (6.6%)	18 (19.1%)	–	–
Contracted research organization	–	34 (36.2%)	17 (73.9%)	19 (82.6%)
Pharmaceutical company	6 (1.7%)	2 (2.1%)	–	–
Other type private company	5 (1.4%)	3 (3.2%)	4 (17.4%)	3 (13.1%)
Total (n)	362	94	23	23

### Participants’ opinions

#### Never worked

**Fig. 1:**
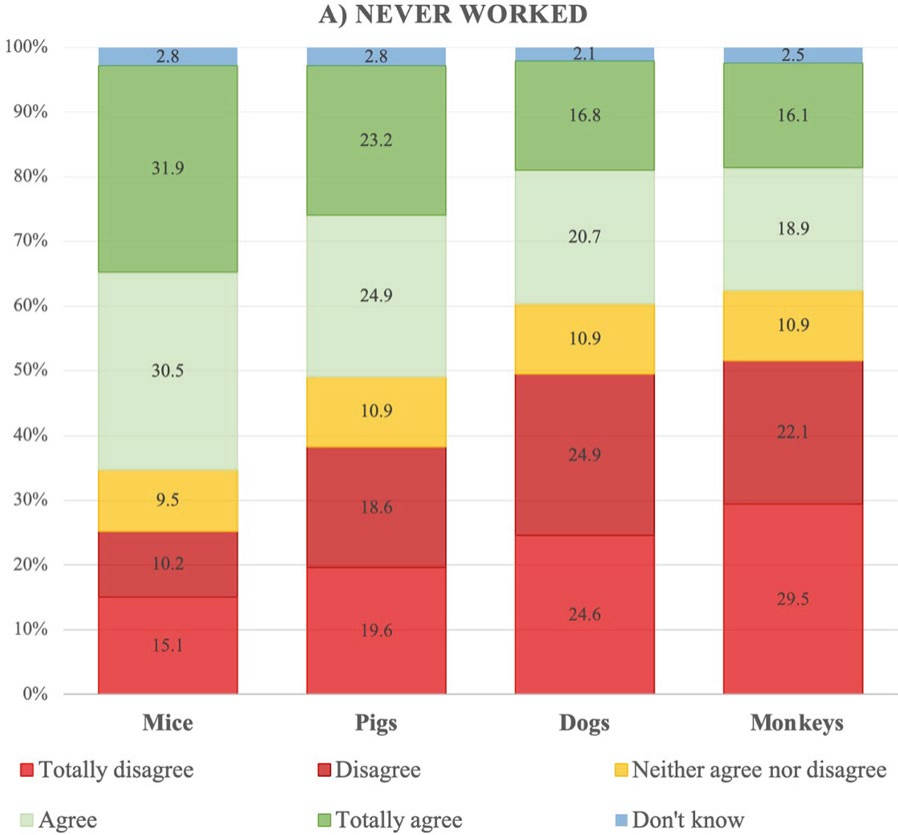
Opinions held by participants who never had worked with laboratory animals regarding research carried out on mice, pigs, dogs or monkeys, providing it produces new information about human health problems, in percentages

Of those participants who had never worked with laboratory animals, more than half agreed or totally agreed with conducting research on mice if it produced new information about human health problems, and just above 25% disagreed or totally disagreed. In relation to research on pigs, almost half agreed or totally agreed, and just under 40% disagreed or totally disagreed. In the case of dogs, these percentages were reversed, with almost half of the participants in this group disagreeing or totally disagreeing with carrying out research on dogs and less than 40% agreeing or totally agreeing. The results for monkeys were very similar, with just over half disagreeing or totally disagreeing and less than 40% agreeing or totally agreeing (Fig. 1). Furthermore, participants’ answers were consistent across different species (Table 5).

**Table 5:** Correlation matrix of the opinions held by participants who had never worked and those who used to work with laboratory animals regarding research carried out with different species

	Never worked				Used to work			
	Mice	Pigs	Dogs	Monkeys	Mice	Pigs	Dogs	Monkeys
Mice	–				–			
Pigs	0.76***	–			0.64***	–		
Dogs	0.87***	0.88***	–		0.31*	0.69***	–	
Monkeys	0.72***	0.85***	0.86***	–	0.50***	0.77***	0.58***	–

^*^p < .05, ***p < .001

The chi-square analysis revealed gender differences, with women who had never worked with laboratory animals disagreeing more than their male counterparts with carrying out research on mice (X^2^_(15)_ = 32,84, *p* = 0.005, Cramer’s V = 0.20), pigs (X^2^_(15)_ = 35.75, *p* = 0.002; Cramer’s V = 0.20), dogs (X^2^_(15)_ = 47.65, *p* < 0. 0001; Cramer’s V = 0.24) and monkeys (X^2^_(15)_ = 27.49, *p* = 0. 025; Cramer’s V = 0.18). No differences were observed in terms of age range or education.

#### Used to work

**Fig. 2:**
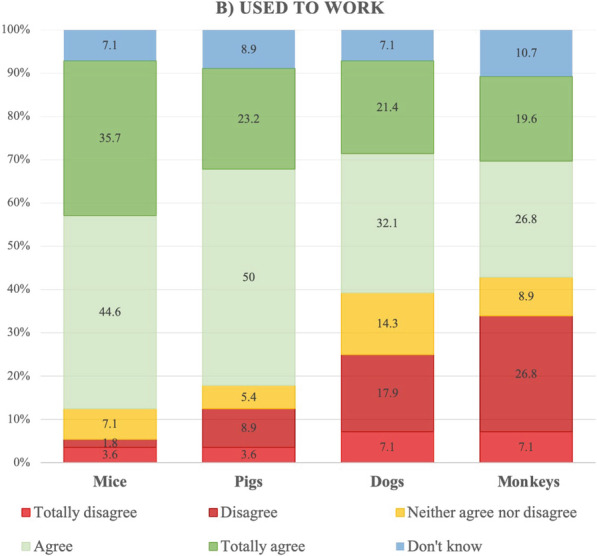
Opinions held by participants who used to work with laboratory animals regarding research carried out on mice, pigs, dogs or monkeys, providing it produces new information about human health problems, in percentages

Participants who had stopped working with laboratory animals mostly agreed or totally agreed with the use of mice and pigs for research purposes. Although this percentage was lower for the other species studied, around half of the participants in this group agreed or strongly agreed with the use of dogs and monkeys. The percentage of those who disagreed or totally disagreed with the use of monkeys and dogs was higher than for pigs and mice (Fig. 2). In this group also, participants’ answers were consistent across species, although the correlations were lower than among participants who had never worked with laboratory animals (Table 5).

The chi-square analysis indicated that men who used to work with laboratory animals disagreed more than their female counterparts with conducting research on mice (X^2^_(10)_ = 22.77, *p* = 0.012, Cramer’s V = 0.45) and pigs (X^2^_(10)_ = 21.72, *p* = 0.017; Cramer’s V = 0.4), although no differences were observed in relation to research on dogs or monkeys, or in terms of age range or education.

#### Currently working

**Fig. 3:**
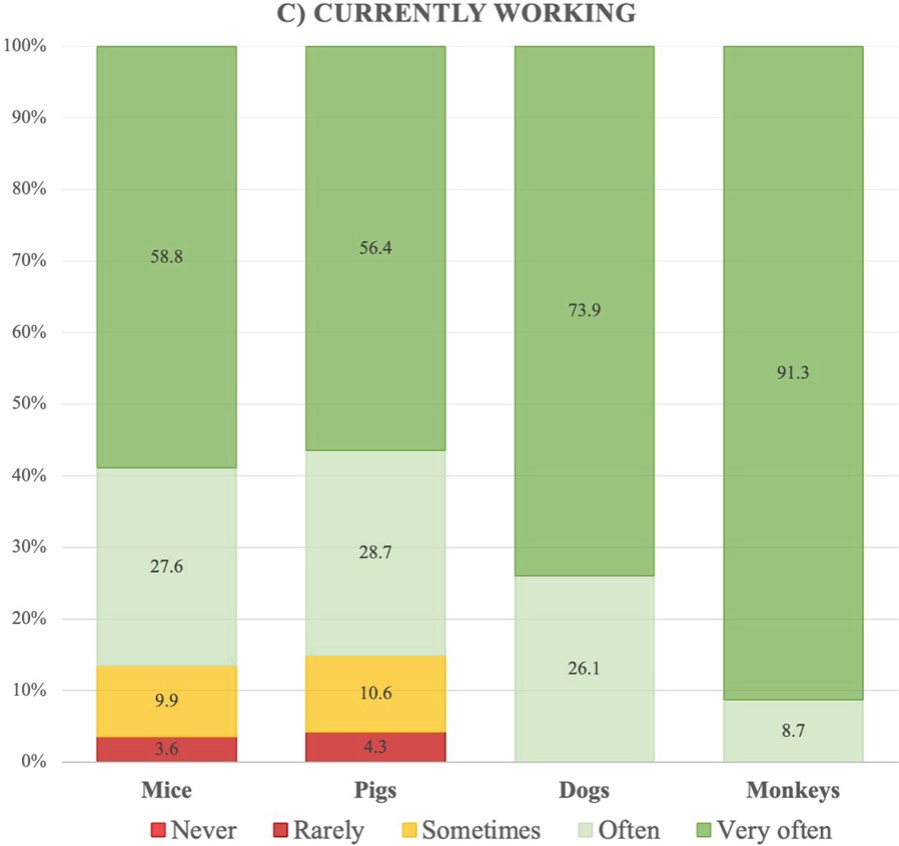
Representation of how often participants thought their work with mice, pigs, dogs or monkeys was justified, in percentages

Most participants working with mice and pigs at the time of the study often or very often thought that their work with laboratory animals was justified, with this percentage being 100% among those working with dogs and monkeys (Fig. 3). No differences were observed in terms of gender, age or education.

**Fig. 4:**
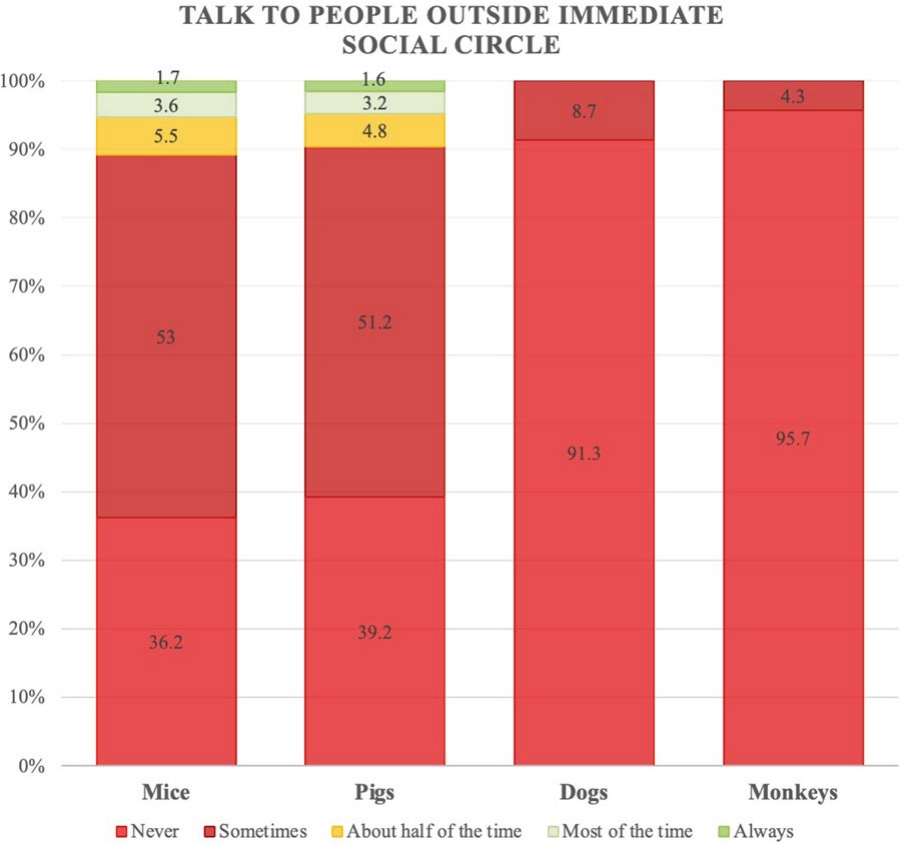
Representation of how often participants talk about their work with people outside their immediate social circle, in percentages

Finally, the chi-square test of independence revealed a significant moderate association between species and talking to people outside one’s immediate social circle about one’s job (X^2^_(12)_ = 61.9, *p* < 0.001; Cramer's V = 0.203). More than 90% of participants working with dogs or monkeys said they never talked about their job, whereas this figure was just under 40% for those working with mice or pigs (Fig. 4).

## Discussion

Animal research in biomedical science remains a controversial topic. In a previous study, we observed that people working with laboratory rodents were cautious and rarely talked about their job, a finding that suggests that animal testing is considered a sensitive issue in Spain [10]. In this study, our aim was to explore what people thought about the use of mice, pigs, dogs and monkeys in biomedical research, in accordance with whether or not they had ever worked with these animals. To this end, we invited people who had never worked with laboratory animals and people who used to work with them to answer the same questions as those posed in the 2010 Eurobarometer [8]. We also invited people who were currently working with the aforementioned species to state whether or not they believed their work with animals was justified. We included pigs in our survey because they are currently considered an optimal species for hosting chimeric human organ formation [16] and because, according to the latest statistical reports on the use of animals for scientific purposes in the European Union [17] and Spain [18], their use has increased over recent years.

Our results revealed that the phylogenetic scale is an important factor in people’s opinions of the use of certain species for research purposes. Among those who had never worked with animals, the number of participants who were against the use of monkeys and dogs was twice as high as the number of those who were against the use of mice. We observed the same trend in the group of those who used to work with animals, with the percentage of those who were against the use of dogs or monkeys being at least five times higher than that of those who were against the use of mice. Moreover, opinions were mostly consistent across different species, with participants who disagreed with the use of mice also disagreeing with the use of other species. These results support the assumption that there is a difference in moral status between at least some larger mammals (e.g., non-human primates, dogs, and pigs) and at least some smaller mammals (e.g., mice) [19]. The differences observed here between the use of pigs and the use of dogs and monkeys may be due to the fact that, in Spain, pigs are considered farm animals. In 2020, Spain was the second largest producer of pork in Europe and the fourth largest in the world [20]. Moreover, in a recent study, participants were found to feel more moral concern for companion animals (e.g., dogs) and appealing wild animals (e.g., chimps) than for food animals (e.g., pigs) [21].

We observed a contradictory gender effect. In the group that had never worked with animals, those who identified as women were more likely to object to animal use than those who identified as men. This finding is consistent with those reported by previous studies [7, 22]. However, in the group of participants who used to work with animals, the opposite effect was observed. Unfortunately, the small size of our sample precludes the possibility of suggesting an explanation for this.

Working with laboratory animals can bring satisfaction, but it can also result in workplace stress [23]. We cannot dismiss the possibility of this being the factor motivating the change in profession reported by most of the participants in our study who no longer worked with laboratory animals. What we did find in our previous study was that the burnout observed among biomedical PhD students in Spain was not related solely to their work with laboratory animals [24]. In Spain, researchers are particularly vulnerable due to the unstable nature of scientific careers and the strong impact of different gender factors, such as motherhood and work-life balance [25]. Empathy and ethical issues were also reasons given by our participants for changing career paths. It is unfortunate that we were not able to recruit a larger sample, as this would perhaps have enabled us to determine the weight of each factor in the final decision to stop working with laboratory animals.

The vast majority of participants who were working with laboratory animals at the time of the study worked with mice, and much fewer worked with large animals. This is consistent with the statistics published on the use of animals in research [18]. Overall, participants believed that the use of animals in their work was justified, although they also said they did not talk about it with people outside their immediate social circle. This attitude was much more prevalent in the case of participants working with dogs and monkeys. Most worked in contracted research organizations, which often work on pharmaceutical toxicity studies, in which the use of two species—a rodent and a non-rodent—is generally expected [26, 27]. These studies are usually required to provide safety data to support clinical development and licensing registration for potential new pharmaceuticals, and the most common non-rodent species used are dogs and non-human primates [28]. This year, a petition to stop the use of Beagles in a toxicity study obtained more than one million signatures in Spain [29]. One month earlier, the first transplant of a genetically-modified pig heart into a human was announced [30], without provoking such a negative response.

The present study has certain limitations. First, we did not ask people working with laboratory animals what they thought about the use of animals in research because we focused more on whether or not they felt their work was justified. We cannot, therefore, compare their responses with those given by the other groups or extrapolate our data to the general Spanish population. Second, the percentage of women and people with a higher education level in our sample was greater than in Spanish society at large, thereby rendering the results less representative of the general population.

## Conclusions

Our study provides evidence that the phylogenetic scale is an important factor influencing people’s opinions of the use of animals in research. Specifically, our findings suggest that there is a difference in moral status between monkeys and mice, and between companion animals (dogs) and farm animals (pigs). The results presented here serve to support the idea that working with laboratory animals is a sensitive issue in Spain.

## Methods

### Participants and procedure

Participants who had never worked (a) and those who no longer worked (b) with laboratory animals were recruited by WhatsApp or e-mail between January 3, 2022 and March 3, 2022. Participants currently working (c) with laboratory animals were recruited by email through a mailing list provided by the Spanish Society for Laboratory Animal Science (SECAL-L) between December 1, 2020 and May 31, 2021. The study was restricted to people over 20 years of age living in Spain. In a cover letter attached to the questionnaire, participants were informed that the survey data would be used for scientific purposes and that they would remain anonymous. All participants gave their voluntary informed consent prior to completing a short 5-min online questionnaire (Google Drive platform). The study was conducted in accordance with the guidelines established by the Declaration of Helsinki. All procedures and informed consent protocols were approved by the Ethics Committee for Human-Related Research (CEISH) of the University of the Basque Country (UPV/EHU); M10/2020/222, M10/2021/136 and M10/2021/365.

### Questionnaire

The questionnaire gathered personal information from participants, including gender, age range, and education. Participants who had previously worked with laboratory animals (b) were also asked about their past institution, job category, years and hours/week spent working with laboratory animals, and their reason(s) for no longer working with them: *allergies, change of research project, change of professional career, salary, ethical issues, empathy towards animals* or *working hours*; space was also provided for participants to write a more detailed response. Participants currently working with laboratory animals (c) were asked about their current institution, job category, years working with laboratory animals and hours/week worked.

Next, participants who had never worked (a) and those who had previously worked with laboratory animals (b) were asked to respond to the following statement: “Scientists should be allowed to do research on animals such as (mice/pigs/dogs/monkeys) if it produces new information about human health problems”; *totally agree, agree, neither agree nor disagree, disagree, totally disagree,* or *don’t know* [8]. Participants currently working with mice, pigs, dogs or monkeys (c) were asked to respond to the statement: “In the last 30 days, how often have you thought that your work with laboratory animals is justified?”; *never, rarely, sometimes, often* or *very often*. We also asked how often they talked to people outside their immediate social circle about their work with laboratory animals; *never, sometimes, about half of the time, most of the time* or *always*.

### Statistical data analysis

All statistical analyses were performed with the Jamovi (1.16.15) and SPSS (Statistics 24) software packages. Frequency (%) and distribution—mean ± standard deviation (SD)—statistics were used to describe the sample. Opinion consistency across species was analyzed using Spearman’s correlation; ≤ 0.3 (weak effect), 0.3—≤ 0.5 (moderate effect), 0.5—≤ 0.7 (substantial effect) and > 0.7 (very strong effect). Chi-square tests were performed to analyze associations between opinions and other variables (gender, age and education) and, if the results were significant, the adjusted residuals were calculated. Cramer’s V was used to calculate effect size; ≤ 0.2 (weak effect), 0.2—≤ 0.4 (moderate effect), 0.4—≤ 0.6 (relatively strong effect) and > 0.6 (strong effect). The level of significance was set to *p* < 0.05. There was no missing data.

## Supplementary Information

**Additional file 1.** **Supplementary Table 1.** Participants by Spanish Autonomous Community.

## Data Availability

Data of the study will we available upon reasonable request to the corresponding author.

## Abbreviations

3Rs: Replacement, reduction and refinement
CEISH: Ethics Committee for Human-Related Research
ECI: European Citizens’ Initiative
EMA: European Medicines Agency
NCad: Netherlands National Committee for the protection of animals used for scientific purposes
SECAL: Spanish Society for Laboratory Animal Science

## References

[1] Dhai A (2014). The research ethics evolution: from Nuremberg to Helsinki. S Afr Med J.

[2] Russell WMS, Burch RL (1959). The principles of humane experimental technique.

[3] Bert B, Chmielewska J, Hensel A, Grune B, Schönfelder G (2016). The animal experimentation quandary: stuck between legislation and scientific freedom: more research and engagement by scientists is needed to help to improve animal welfare without hampering biomedical research. EMBO Rep.

[4] Kolar R (2002). ECVAM: desperately needed or superfluous? An animal welfare perspective. Altern Lab Anim.

[5] De la Fuente M, Souto A, Caselli C, Schiel N (2017). People’s perception on animal welfare: why does it matter?. Ethnobiol Conserv.

[6] Knight S, Barnett L (2008). Justifying attitudes toward animal use: a qualitative study of people's views and beliefs. Anthrozoös.

[7] Ormandy EH, Schuppli CA (2014). Public attitudes toward animal research: a review. Animals (Basel).

[8] EC. European Commission Special Eurobarometer 340—Science and Technology. 2010. https://data.europa.eu/data/datasets/s806_73_1_ebs340?locale=en. Accessed 26 Apr 2022.

[9] Pejman N, Kallas Z, Dalmau A, Velarde A (2019). Should animal welfare regulations be more restrictive? A case study in eight European Union countries. Animals (Basel).

[10] Goñi-Balentziaga O, Ortega-Saez I, Vila S, Azkona G (2021). Working with laboratory rodents in Spain: a survey on welfare and wellbeing. Lab Anim Res.

[11] ECI. Stop Vivisection. 2015. http://www.stopvivisection.eu/en. Accessed 26 Apr 2022.

[12] NCad. NCad opinion Transition to non-animal research 2016. https://www.ncadierproevenbeleid.nl/documenten/rapport/2016/12/15/ncad-opinion-transition-to-non-animal-research. Accessed 26 Apr 2022.

[13] Revill J. Swiss reject ban on animal testing in referendum. 2022. https://www.reuters.com/world/europe/switzerland-vote-becoming-first-nation-ban-animal-testing-2022-02-13/. Accessed 26 Apr 2022.

[14] Kutschenko LK, Hagen K, Schnieke A, Thiele F (2012). Relevant similarity in the light of biomedical experimentation. Large animals as biomedical models: Ethical, societal, legal and biological aspects.

[15] Heeger R, Hagen K, Schnieke A, Thiele F (2012). Experimenting on animals: When does their size matter morally?. Large animals as biomedical models: Ethical, societal, legal and biological aspects.

[16] Morata Tarifa C, López Navas L, Azkona G, Sánchez PR (2020). Chimeras for the twenty-first century. Crit Rev Biotechnol.

[17] EC. Summary Report on the statistics on the use of animals for scientific purposes in the Member States of the European Union and Norway in 2018. 2021. https://ec.europa.eu/environment/chemicals/lab_animals/pdf/SWD_%20part_A_and_B.pdf. Accessed 26 Apr 2022.

[18] MAPA. Informe sobre usos de animales en experimentación y otros fines científicos, incluyendo la docencia. 2019. https://www.mapa.gob.es/es/ganaderia/temas/produccion-y-mercados-ganaderos/informedeusodeanimalesen2019_tcm30-550894.pdf. Accessed 26 Apr 2022.

[19] Walker RL, Eggel M (2020). From mice to monkeys? Beyond orthodox approaches to the ethics of animal model choice. Animals (Basel).

[20] MAPA. El sector de la carne en cifras. 2020. https://www.mapa.gob.es/es/ganaderia/estadisticas/indicadoreseconomicossectorporcino2020_tcm30-379728.pdf. Accessed 26 Apr 2022.

[21] Krings VC, Dhont K, Salmen A (2021). The moral divide between high- and low-status animals: the role of human supremacy beliefs. Anthrozoös.

[22] Sandgren EP, Streiffer R, Dykema J, Assad N, Moberg J (2020). Attitudes toward animals, and how species and purpose affect animal research justifiability, among undergraduate students and faculty. PLOS ONE.

[23] Murray J, Bauer C, Vilminot N, Turner PV (2020). Strengthening Workplace Well-Being in Research Animal Facilities. Front Vet Sci.

[24] Goñi-Balentziaga O, Vila S, Ortega-Saez I, Vegas O, Azkona G (2021). Professional quality of life in research involving laboratory animals. Animals (Basel).

[25] OMCI. Estudio sobre la situación de las jóvenes investigadoras en España. Secretaría General Técnica del Ministerio de Ciencia e Innovación. 2021. https://www.culturaydeporte.gob.es/dam/jcr:875ee2f6-37e7-494e-9767-6434f7ee1b06/informe-jovenes-investigadoras-esp.pdf. Accessed 26 Apr 2022.

[26] ICHM3(R2). Nonclinical safety studies for the conduct of human clinical trials and marketing authorization for pharmaceuticals. In: International Conference on Harmonisation (ICH). 2009. https://www.ema.europa.eu/en/ich-m3-r2-non-clinical-safety-studies-conduct-human-clinical-trials-pharmaceuticals. Accessed 26 Apr 2022. 20349552

[27] ICHS9. Nonclinical evaluation for anticancer pharmaceuticals. In: International Conference on Harmonisation (ICH). 2010. https://www.ema.europa.eu/en/ich-s9-non-clinical-evaluation-anticancer-pharmaceuticals. Accessed 26 Apr 2022.

[28] Prior H, Haworth R, Labram B, Roberts R, Wolfreys A, Sewell F (2020). Justification for species selection for pharmaceutical toxicity studies. Toxicol Res (Camb).

[29] Change.org. Salvar a los 38 cachorros Beagle del laboratorio Vivotecnia de su ejecución. 2022. https://www.change.org/p/universidad-de-barcelona-salvar-a-los-38-cachorros-beagle-del-laboratorio-vivotecnia-de-su-ejecuci%C3%B3n. Accessed 26 Apr 2022.

[30] Kotz D. University of Maryland School of Medicine Faculty Scientists and Clinicians Perform Historic First Successful Transplant of Porcine Heart into Adult Human with End-Stage Heart Disease. 2022. https://www.medschool.umaryland.edu/news/2022/University-of-Maryland-School-of-Medicine-Faculty-Scientists-and-Clinicians-Perform-Historic-First-Successful-Transplant-of-Porcine-Heart-into-Adult-Human-with-End-Stage-Heart-Disease.html. Accessed 26 Apr 2022.

