# Supplementary material for: A survey on the use of mice, pigs, dogs and monkeys as animal models in biomedical research in Spain
Source: Lab Anim Res. 2022 Jun 2;38:14. doi: 10.1186/s42826-022-00124-5 (PMC9161537; doi:10.1186/s42826-022-00124-5)
Supplement: Supplementary file 1 — Additional file 1. Supplementary Table 1. Participants by Spanish Autonomous Community. [file 42826_2022_124_MOESM1_ESM.docx]

**Supplementary Table 1.** Participants by Spanish Autonomous Community.

|  | **Never worked** | **Used to work** | **Currently working** | **Total** |
| --- | --- | --- | --- | --- |
|  | n (%) | | |  |
| Andalucía | 12 (4.2%) | 3 (5.4%) | 36 (7.7%) | **51 (6.3%)** |
| Aragón | 4 (1.4%) | - | 4 (0.9%) | **8 (1%)** |
| Asturias | 1 (0.4%) | - | 2 (0.4%) | **3 (0.4%)** |
| Balearic Islands | - | - | 1 (0.2%) | **1 (0.1%)** |
| Basque Country | 175 (61.5%) | 26 (46.4%) | 68 (14.6%) | **269 (33.3%)** |
| Canary Islands | 1 (0.4%) | - | 3 (0.6%) | **4 (0.5%)** |
| Cantabria | 7 (2.5%) | 1 (1.8%) | - | **8 (1%)** |
| Castilla La Mancha | 2 (0.7%) | - | 15 (3.2%) | **17 (2.1%)** |
| Castilla León | 6 (2.1%) | - | 2 (0.4%) | **8 (1%)** |
| Catalonia | 57 (20%) | 15 (26.8%) | 182 (39.1%) | **254 (31.5%)** |
| Comunidad Valenciana | 7 (2.5%) | 2 (3.6%) | 28 (6.0%) | **37(4.6%)** |
| Galicia | 4 (1.4%) | 2 (3.6%) | 26 (5.6%) | **32 (4%)** |
| Madrid | 3 (1.1%) | 6 (10.7%) | 89 (19.1%) | **98 (12.1%)** |
| Murcia | - | - | 2 (0.4%) | **2 (0.2%)** |
| Navarra | 6 (2.1%) | 1 (1.8%) | 8 (1.7%) | **15 (1.9%)** |
